# Merging Proline:Xylitol Eutectic Solvent in Crosslinked Chitosan Pervaporation Membranes for Enhanced Water Permeation in Dehydrating Ethanol

**DOI:** 10.3390/membranes13040451

**Published:** 2023-04-21

**Authors:** Roberto Castro-Muñoz, Maksymilian Plata-Gryl, Grzegorz Boczkaj

**Affiliations:** 1 Faculty of Civil and Environmental Engineering, Department of Sanitary Engineering, Gdansk University of Technology, 11/12 Narutowicza St., 80-233 Gdansk, Poland; 2 Tecnologico de Monterrey, Campus Toluca, Av. Eduardo Monroy Cárdenas 2000 San Antonio Buenavista, Toluca de Lerdo 50110, Mexico; 3 Advanced Materials Center, Gdansk University of Technology, 11/12 Narutowicza St., 80-233 Gdansk, Poland

**Keywords:** proline:xylitol, deep eutectic solvents, chitosan, water-ethanol, water transport, natural deep eutectic solvents (NADESs)

## Abstract

The scope of this research aims at merging a new deep eutectic mixture (DES) into a biopolymer-based membrane for a pervaporation application in dehydrating ethanol. Herein, an L-proline:xylitol (at 5:1) eutectic mixture was successfully synthesized and blended with chitosan (CS). A complete characterization of the hybrid membranes, in terms of morphology, solvent uptake, and hydrophilicity, has been conducted. As part of their applicability, the blended membranes were assayed for their ability to separate water from ethanolic solutions by means of pervaporation. At the highest temperature (50 °C), a water permeation of ca. 0.46 kg m^−2^ h^−1^ was acquired, representing a higher permeation than the pristine CS membranes (ca. 0.37 kg m^−2^ h^−1^). Therefore, CS membranes demonstrated an enhanced water permeation thanks to their blending with the hydrophilic L-proline:xylitol agent, making these membranes a good candidate for other separations containing polar solvents.

## 1. Introduction

Pervaporation (PV), as an alternative technique for solvent separation, seeks for the development of a new concept of membranes that may offer not only a superior separation performance but also a more sustainable fabrication pathway. Today, there is a current trend of implementing bio-based materials in the preparation of membranes [1]. In this way, several biopolymers have been proposed for PV membrane fabrication, including sodium alginate [2], poly lactic acid [3], cellulose-based polymers [4], collagen, and chitosan (CS), among others, and this latter biopolymer has been deeply investigated over the recent years [5]. In general, CS, as a hydrophilic membrane material, represents an exceptional candidate for the separation of polar compounds from other less-polar (or non-polar) solvent molecules. To date, several strategies, such as crosslinking [6], polymer blending [7], and nanomaterial blending [8,9], have been proposed to overcome the main constraints of CS membranes in terms of swelling and mechanical, chemical, and thermal stability, also keeping in mind a possible improvement in the separation efficiency of the membranes. To some extent, the embedding of nanomaterials into polymer phases seems to offer great advances for renewable energy applications [10,11,12]. However, a main criterion to be considered during the blending of external agents with CS is the biocompatibility and good interaction among both phases. In light of the compatible external agents or additives with the CS phase, deep eutectic solvents (DESs) have been referred to as exceptional agents for boosting key CS properties, such as mechanical, morphological and separation features [13,14,15]. As reported elsewhere, a typical DES implies the mixture of two different organic compounds, namely HBA (hydrogen-bond acceptor) and HBD (hydrogen-bond donor), which are self-assembled thanks to H-bonding. In principle, both substances, such as amide, amino acid, sugar, alcohol, amine, carboxylic acid (as HBA), and quaternary ammonium salt (as HBD), are cheap and non-toxic. So far, DESs, apart from tentatively meeting the key requirements of the 12 principles of ‘green chemistry’, have been implemented in a number of applications of chemistry, materials science, and analytical chemistry [16,17,18]. In this field, a high interest relates to a so-called Natural DESs (NADESs), which are obtained from components of natural origin [19].

In membrane separation, a hydrophilic DES can introduce the enhanced transport of polar molecules, which is ascribed to their boosted adsorption and diffusion of the chemical functionalities of the eutectic mixture [20,21,22]. To some extent, the matching of a eutectic phase and CS still remains a challenge since not all DESs are chemically compatible with polymers, causing a lack of miscibility. Therefore, as a starting point, hydrophilic eutectic solvents tend to display a good affinity with CS, resulting in homogenous polymer films [14]. In this work, we launch a new hydrophilic and water-soluble eutectic solvent based on a protonated L-proline:xylitol (at 5:1) eutectic mixture, which was later blended into CS and then chemically crosslinked with a crosslinker agent (glutaraldehyde). The new DES as a green additive was evaluated for its chemical blending and its effect on CS structure. The resulting membrane films were morphologically studied using scanning electron microscopy (SEM) and atomic force microscopy (AFM), hydrophilicity (in terms of water contact angle), FTIR spectroscopy, and solvent uptake. Moreover, the membrane films were also studied in a hydrophilic PV laboratory test by separating water/ethanol (10/90) mixtures. PV tests were evaluated at different operating temperatures ranging from 20 to 50 °C.

## 2. Materials and Methods

### 2.1. Reactants and Materials

L-proline (purity ≥ 98%, Sigma Aldrich, Poznan, Poland), xylitol (pure, WarChem, Warsaw, Poland), hydrochloric acid (analytical reagent, POCH S.A., Gliwice, Poland) and GA (grade II, 25 wt.%) were acquired. The chitosan polymer (medium molecular weight) was purchased from Sigma Aldrich.

### 2.2. Hydrophilic DES Synthesis

A protonated-L-proline:xylitol, presenting a molar ratio (5:1), was successfully produced. Briefly, 10 g of L-proline and 86.86 mL of HCl 1M were mixed at 1000 rpm (70 °C) until we obtained a transparent solution. Next, 2643 g of xylitol was incorporated into the mixture. The residual water was finally eliminated via a rotary vacuum evaporator (Rotavapor R-300 with a V-300 vacuum pump, BUCHI, Essen, Germany).

### 2.3. Membrane Preparation

A traditional dense-film casting method and solvent evaporation were used to prepare the membrane films. Briefly, 1.5 wt.% of polymer went into an acidic solution with continuous mixing for a day. After this, the eutectic mixture, ca. 5 wt.% in relation to the polymer percentage, was blended into the polymer dope dissolution. The DES percentage was determined previously in other related investigations [14,23]. The final dissolution was again mixed over a certain time (4 h) before applying the chemical in-situ crosslinking. For this latter treatment, 100 µL of glutaraldehyde, with a subsequent 100 µL of HCl, was added to the final dope mixture. After a 15 min mixing period, the resulting dope mixtures were finally cast onto Petri dishes. The membranes were exposed to solvent evaporation over 48h. It is important to highlight that the membranes presented clear homogeneity and an average thickness ca. 30 μm, as measured by the SEM technique.

### 2.4. Characterization of Membrane Films

Scanning electron microscopy (SEM) and atomic force microscopy (AFM): Two different microscopy techniques were used to study the membranes’ morphology. For SEM, the samples were initially treated with gold layer sputtering with a final layer of 10 nm. Then, the treated samples were observed using an FEI Quanta FEG 250 SEM—field emission gun scanning electron microscope. For cross-section views, the membranes were subjected to liquid nitrogen to intentionally produce a fracture. To observe the membrane’s roughness and surface, a Nanosurf EasyScan 2 was used in contact mode using silicon tips (AppNano—SICON series), with a 10 nN constant force [20].

FTIR spectroscopy: For this analysis, membranes were investigated using a Nicolet iS10 spectrometer (from Thermo Fisher Scientific, Poznan, Poland), which possesses a DTGS detector and a Golden Gate diamond ATR accessory. The spectrum was recorded as a 4000 to 400 cm^−1^ wave number in a 16 cm^−1^ resolution.

Water contact angle (CA): The CA analysis was performed with a sessile drop using a goniometer OCA15 (Data Physics). Here, the data were reported as the average and standard deviation (SD), as a result of five measurements.

Uptake: The solvent uptake was explored for pure ethanol and ethanolic solutions containing 10 to 50 wt.% water. Commonly, membranes are initially weighed (Wd) and then immersed into the solvent solutions (at room temperature for approximately 2 days). After solvent immersion, the remaining solvent was directly cleaned from the wet membrane films. Fastly, the membrane films were weighed (W_w_) using a digital balance (Gibertini, Crystal 500, Novate Milanese, Italy, accuracy 0.001 g). The uptake values were estimated by weight difference, as below [21]:

(1) $$\mathrm{Uptake}\;(\%)=\frac{W_{w}{-W}_{d}}{W_{d}}\cdot 100$$

### 2.5. Pervaporation Separation Experiments for Binary Mixtures

**Figure 1:**
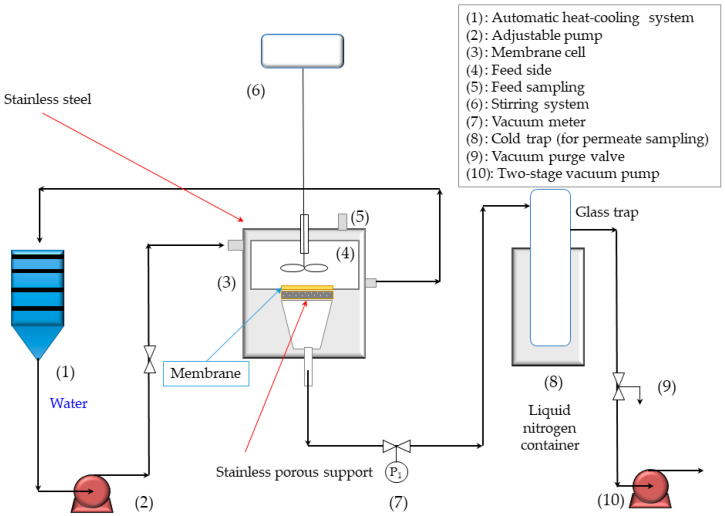
Graphical depiction of the experimental set-up used in PV experiments.

The PV analysis was studied in a lab-scale system, as reported in former studies [24]. Figure 1 shows a graphical depiction of the PV set-up used in this work. Experimentally, ethanolic model solutions (10 wt.% water in ethanol) were prepared and used for different PV experiments, in which the temperature varied from 20 to 50 °C. In this experimentation, a vacuum pressure was applied (constant 1 mbar, RVpro 4 vacuum pump, Welch Vacuum Products, Niles, IL, USA). In the PV system, the permeating vapor was captured using a glass trap, which was immersed in liquid nitrogen. PV runs were carried out in a continuous operation for 4h. The total permeate flux (J) was calculated as a relation of total weight of permeate (Q), membrane area (A) and operating time (t), as given by Equation (2):

(2) $$J=\frac{Q}{A\;\cdot \;t}$$

The partial flux (J_i_) for each component i results as the product of multiplying its weight fraction (y_i_) and the total flux (J), as denoted below:

(3) $$J_{i}{=Y}_{i}\cdot J$$

To evaluate the membrane’s separation efficiency, the separation factor (α) was used as the parameter, which relates the water and ethanol weight fractions at permeate and feed sides, as in Equation (4) [23]:

(4) $$\alpha =\frac{y_{\mathrm{water}}/y_{\mathrm{ethanol}}}{x_{\mathrm{water}}/x_{\mathrm{ethanol}}}$$

The permeate and feed compositions were determined via gas chromatography (GC) with an Autosystem XL gas chromatograph with a flame ionization detector (FID) and split/splitless injector (Perkin Elmer, Waltham, MA, USA). Separation was performed using a 60.0 m × 0.32 mm ID × 1 μm (DB-624) capillary column (Agilent, Santa Clara, CA, USA) using nitrogen as carrier gas.

### 2.6. Mass Transfer Evaluation in Prepared Membranes

Mass-transfer resistance in the studied system was evaluated by the resistance in series theory assuming the solution-diffusion mechanism [25,26]. The theory predicts that the mass-transfer coefficient K_overall,i_ for a component i can be described by the sum of the system components resistances, as follows:

(5) $$\frac{1}{K_{\mathrm{overall},i}}=\frac{1}{K_{l,i}}+\frac{1}{K_{m,i}}=\frac{P_{i}^{\mathrm{sat}}\gamma_{i}}{k_{L,i}\rho }+\frac{\sigma }{P_{i}}$$

where K_l,i_ and K_m,i_ represent the mass-transfer coefficients of component i in the liquid phase and in the membrane, respectively. $P_{i}^{\mathrm{sat}}$ , y_i_, k_L,i_, ρ, σ and P_i_ are the saturated vapor pressure of a component i, its molar fraction in the permeate, its mass-transfer coefficient in the feed, the density of the feed, the membrane thickness, and the permeability of a component i through a membrane layer, respectively. Details of the calculations can be found in our previous works [24,27].

## 3. Results and Discussion

### 3.1. Membrane Characterization

**Figure 2:**
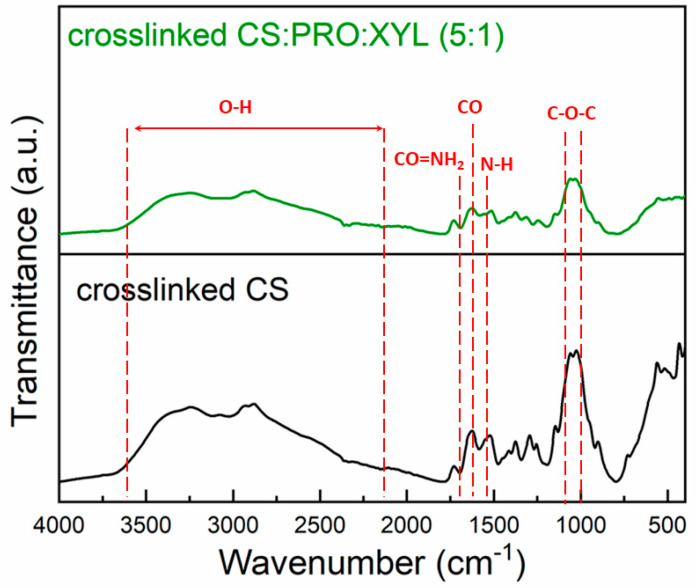
FTIR spectra for pristine crosslinked CS and CS:PRO: XYL membranes.

An important aspect revealing the good blending between the eutectic mixture and CS can be observed through their possible chemical interaction. In this context, the FTIR profiles somehow affirm the effective merging of both phases (see Figure 2). Thanks to the overlapping and the O-H and N-H stretching of chemical functionalities linked to H-bonds, the spectra reveal defined and non-symmetric patterns at 3350 cm^−1^. As found in the literature, CS regularly displays distinctive absorption bands, e.g., C=O stretching in the amide group can be noted at 1600 cm^−1^, while the N-H bending in non-acetylated 2-aminoglucose is seen around 1550 cm^−1^ and the N-H bending in the amide group can be observable in a pretty close range (ca. 1560 cm^−1^) [28]. At 1100 cm^−1^, absorption bands can be visualized corresponding to the antisymmetric stretching of the C-O-C bridge. As for the eutectic mixture (PRO:XYL), L-proline presents an imine as an extra amine group. This second amino group allows us to recognize proline as an imino acid. Peculiarly, since it is known that proline’s three-carbon R-group is fused to the α-nitrogen group [29], proline presents a rotationally constrained rigid-ring. As for xylitol, it belongs to the classification of polyalcohols. It exhibits a broad vibration around 3500 cm^−1^ according to the stretching of the OH groups. In any eutectic mixture, the HBA and HBD are habitually self-assembled due to the H-bonding, leading to a eutectic mixture; this latter interaction can be seen with a firm shifting and broadening between 3700 and 2000 cm^−1^ [30]. When PRO:XYL is blended with CS, a smooth but clear shift on the characteristic CS bands is noted. This latter fact confirms a positive interaction for the PRO:XYL and the polymer, resulting in an acceptable compatibility. In the range of 3700 to 3000 cm^−1^, detectable shifts in the ordinary shapes of the band were found, which were ascribed to the overlapping of the bands and tentatively credited to O-H, N-H and C-O vibrations in chemical groups of both the polymer and PRO:XYL.

CS/DES films have evidenced comparable chemical interactions [13]. It is worth mentioning that the crosslinked membranes containing glutaraldehyde frequently display an absorption shift around 1600–1650 cm^−1^, attributed to imine bonds N=C [31,32]. On the other hand, a slight stretching of about 1540, 1710 and 2900 cm^−1^ may be related to the carbonyl group (C=O) in free-aldehydes and increased C-H stretch, respectively. Additionally, it was noted that aliphatic amino groups in CS were weakened as much as the peak 1100 cm^−1^.

**Figure 3:**
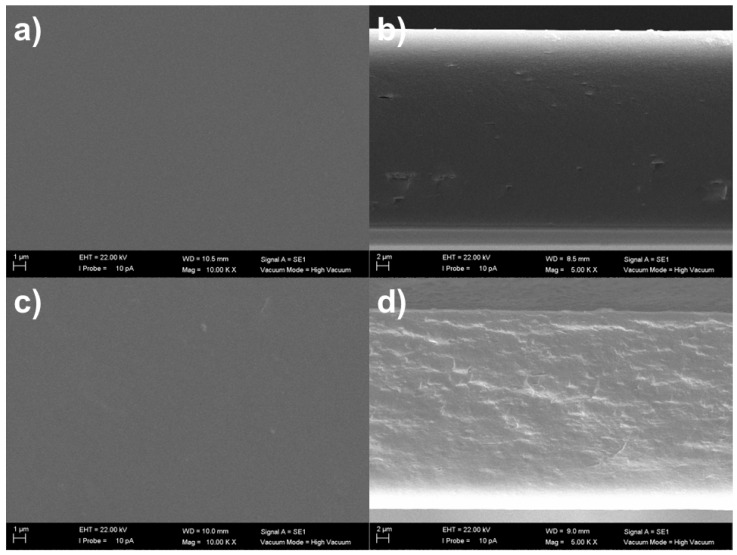
SEM surface and cross-section view images of the fabricated membranes based on CS and L-proline:xylitol DES. (**a**,**b**) crosslinked chitosan and (**c**,**d**) crosslinked chitosan:L-proline: xylitol (CS:PRO:XYL).

As for the morphological characterization, flat and continuous surfaces were seen in both membranes with and without the eutectic mixture (see Figure 3a,c), along with an evident lack of plastic deformation, as is commonly observed in non-porous (or dense) polymer membranes [33]. Regarding the membrane structure in cross-section images, the DES-free CS membrane presented a typical crater-like pattern (Figure 3b), which frequently takes place in deformation induced by freeze-fracture [24,27]. Similar morphology has been acquired in the membranes with PRO:XYL; however, differently from pristine CS membranes, no visible gaps and craters were observed in DES-containing membranes with dense effective structure and non-accumulation of the eutectic mixture (Figure 3d). To some extent, the resulting morphology could be recognized as a proof of good compatibility among the hydrophilic PRO:XYL and CS. When compared with other works reporting DES-containing CS membranes, the CS:PRO:XYL displayed less continuous phases than other similar CS membrane formulations containing different eutectic mixtures, such as L-proline:sulfolane [14] and choline chloride:malonic acid [13].

In this work, it is likely that the incorporation of the DES has unexpectedly contributed to obtaining a less-tight and less-dense structure. Even if it is known that DESs can strategically be used as a pore former additive for porous membrane fabrication [34], our target is to combine and maintain the selected hydrophilic PRO:XYL mixture in the membrane material, allowing us to potentialize the separation properties once present in the membrane film. We believe that the presence of the hydrophilic eutectic mixture affects membrane performance, offering higher permeation rates due to the presence of gaps and craters. Apart from that, DESs can boost the transport of polar molecules thanks to the inherent hydrogen bonds and other specific solute-solvent interactions [18,35].

**Figure 4:**
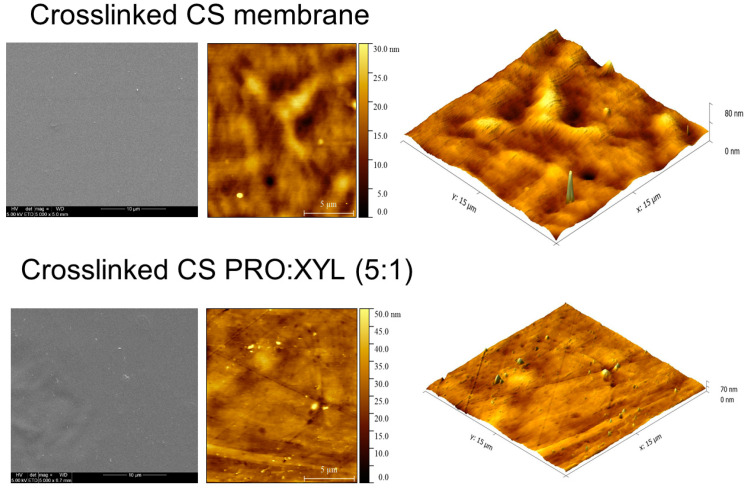
AFM surface and 3D images (15 × 15 µm) of pristine CS (reproduced from [27]) and CS:PRO:XYL membranes.

Potentially, a hydrophilic eutectic mixture based on PRO and XYL has several chemical functionalities, including amino-, hydroxyl-, oxygen- and carbonyl-containing groups, interconnecting with CS. The surface micrograph of the DES-containing CS membranes still demonstrates a smooth, continuous, and defect-free surface and no visible pinholes. On the contrary, it has been demonstrated that choline chloride:malonic acid can turn the smooth surface of CS films into a non-homogenous surface while keeping a dense pattern [13]. Here, the smooth surface was evidenced even when presenting a eutectic mixture, as seen in Figure 4. For instance, the DES-free crosslinked CS membrane had an average root mean square roughness of S_q_ = 4.0 ± 0.5 nm, for which the value becomes lower after the addition of PRO:XYL (ca. S_q_ = 2.0 ± 0.5 nm). This is relevant from the separation point of view since depending on the type of DES phase, the excessive content of the eutectic phase may promote the higher values of roughness in the membranes [15]. In a recent study, it was reported that PRO:GLU-based DESs had also a similar behavior and slightly lowered the roughness from 4 to 3 nm in CS membranes [24]. According to Pontillo et al. [36], the resultant smoothness of CS membranes doped with DES reveals an existing interaction with the chitosan macromolecule.

**Figure 5:**
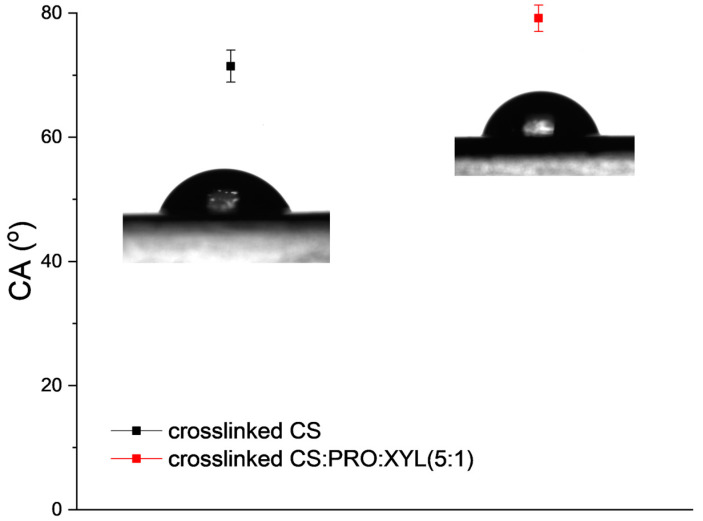
CA values for pristine crosslinked CS (reproduced from [24]) and CS:PRO:XYL membranes.

The low value of roughness in the CS: PRO:XYL membranes allows us to have a perspective of possible hydrophilic membranes. Initially, DES-free crosslinked CS membrane offered a water contact angle of about 70°, as represented in Figure 5. Contact angle measurements were already proved to be effective for characterizing the polarity and hydrophilicity of DESs [37]. This outcome agrees with the literature, where angle values ranging from 74 to 88° have been registered for CS membranes [38,39]. In principle, as for chitosan, the hydrophilic or hydrophobic nature varies as a function of the polymer’s deacetylation degree (DA); for example, a high DA results in highly hydrophilic chitosan membranes, which are ascribed to more amine functionalities available in CS [28], favoring the water permeance thanks to the affinity across the membrane [29]. As for its hydrophilic nature, CS presents hydrophilic functionalities such as -OH and -NH_2_, which are used during the crosslinking; consequently, its hydrophilicity is reduced when crosslinking is performed. Interestingly, the incorporation of the PRO: XYL DES into CS also contributed to the lower hydrophilicity obtained in terms of CA (ca. 79°). To some extent, this latter finding opens the possibility that the polar groups present in the PRO: XYL mixture (such as amino, hydroxyl and carboxyl) are also playing a role in the crosslinking reaction. According to its chemical structure, xylitol is classified as highly polar due to -OH groups being prone to forming hydrogen bonds.

### 3.2. Pervaporation Performance towards Water-Ethanol Model Solutions

#### 3.2.1. Effect of the Operating Temperature on Separation and Permeation

**Figure 6:**
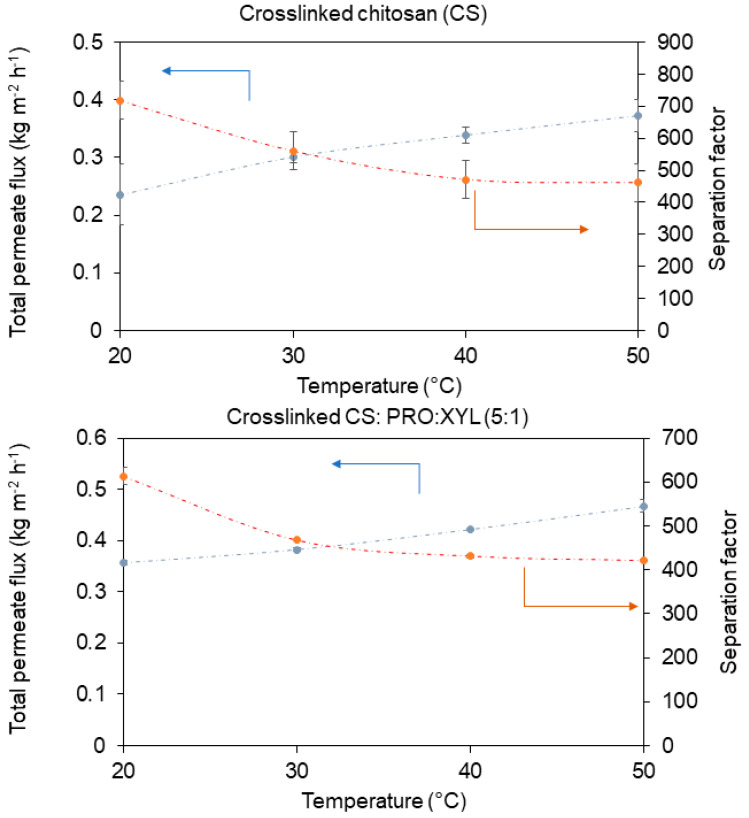
Permeation and separation factor as function of feed temperature (10:90 wt.% water:ethanol, pressure: 1 mbar). The curves are only guiding the eye.

Figure 6 illustrates the behavior of the feed temperature from 20 to 50 °C on both important parameters: permeation and separation efficiency. Initially, a typical increase in permeation flux can be noted in both in the pristine crosslinked membrane and the membrane blended with PRO: XYL. This increasing trend is commonly observed in polymeric membranes, which display substantial flexibility in the polymer chains when the temperature is raised; this thermal motion, usually at higher temperatures, improves the sorption properties of the solvent. This is thereafter translated to better transport among the intermolecular distances of the polymer [40]. For instance, both free and DES-containing membranes showed the highest total permeation at the highest operating temperature, as expected. The PRO: XYL-containing membrane exhibited a total flux of ca. 0.46 kg m^−2^·h^−1^ (see Table S1 for precise PV numerical data), which represents higher permeation than the bare CS membrane with 0.37 kg m^−2^·h^−1^. It is worth mentioning that this eutectic mixture (PRO:XYL) greater favors the permeation across the structure of the CS membrane compared to other blends such as the PRO: GLU CS membrane [24], which showed a maximum permeation of 0.38 kg m^−2^·h^−1^ for water/ethanol separations. Hypothetically, it has been reported that DES-modified CS films with improved water vapor transport can be credited to the penetration of adjacent CS chains by DES molecules, which can result in worsened intramolecular interactions and thus enhance the segmental chain mobility and facilitate the permeation of water vapor molecules at the molecular level [13]. Also, as the water transport in PV membranes involves sorption, diffusion and desorption, the surface structure modified by the hydrophilic DES can then affect both sorption and desorption steps, resulting in changes in the membrane barrier properties [13].

To demonstrate a temperature dependence of the permeate flux, we analyzed the permeation data using the Arrhenius equation and can thus better observe the effect of the temperature in thermally induced processes, as expressed in the following Equation (6).

(6) $${J=J}_{o}\cdot exp\left(-\frac{E_{A}}{R\;\cdot \;T}\right)$$

As found elsewhere, *J_o_* represents the pre-exponential factor, while *E_A_* is the apparent activation energy for permeation and *R*·*T* regards the typical energy term. By mathematically applying logarithms to Equation (5), *E_A_* is calculated as a linear function demonstrating a possible relationship between temperature and permeate; in other words, a total permeation increase can be obtained thanks to an increase in temperature. Thermodynamically, water presented lower *E_A_* values (ca. 5.07 kJ/mol) compared with ethanol (ca. 10.27 kJ/mol) in the bare crosslinked CS membrane, proving its water selectivity. More interestingly, the hydrophilic PRO:XYL blending decreased the *E_A_* value for both water and ethanol (see Table 1); however, a bigger effect was observed in water since the value decreased to 3.02 kJ/mol in the CS:PRO:XYL membrane, while the *E_A_* value for ethanol was less influenced by the DES incorporation (ca. 7.21 kJ/mol). Additionally, it can be pointed out that the operating temperature had a greater impact on water and did not greatly favor the ethanol transport. Notably, the application of this hydrophilic PRO:XYL decreases the energy demanded for the molecules to permeate across the resultant membranes. This agrees with the DES hydrophilicity and its affinity toward more polar compounds (such as water) than ethanol [30].

Analyzing the selective properties of the resultant membranes, Figure 6 presents how the separation factor in the bare CS membrane decreased as a function of temperature. Unfortunately, the separation factor was slightly worsened by incorporating the DES, displaying a value of ca. 613 (at 20 °C). The highest separation factors were acquired with the lower permeation rates, which indeed were obtained at the lowest temperature of operation. The free volume theory establishes that the thermal motion of polymer chains in the amorphous regions enlarge the free volume. It has been noted that when the temperature increases, the frequency and amplitude of the chain jumping also increase, leading to an increase in free volume [31]. Such an increase in free volume may affect the selective properties of the polymer membranes, as thermal motion (and the related increase in free volume) can allow the passage of larger molecules than water (kinetic diameter = 2.6 Å). In this case, ethanol owns a kinetic diameter of approximately 4.3 Å, which can be easily transported if such free volume is enlarged.

**Figure 7:**
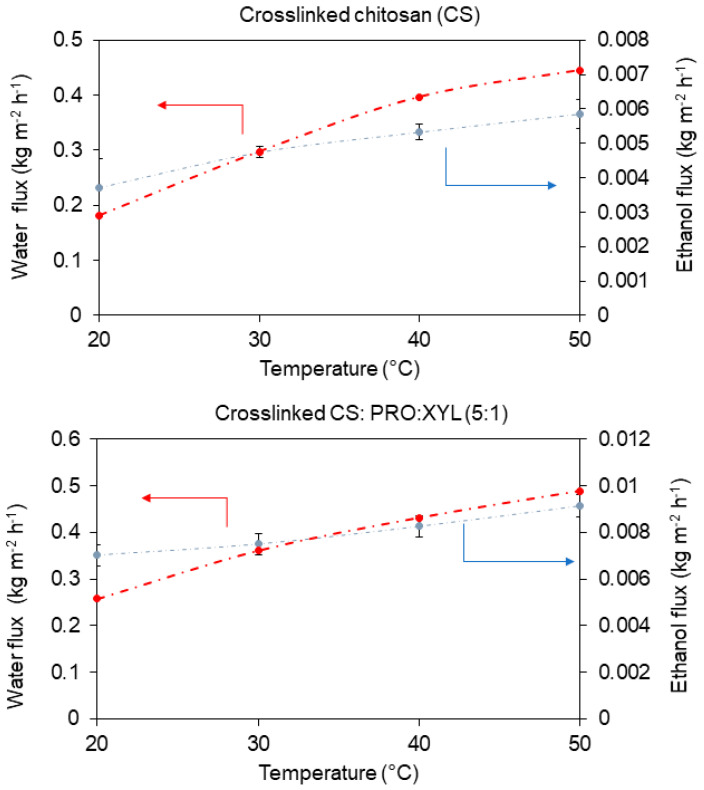
Water and ethanol partial fluxes as a function of operating temperature (10:90 wt.% water-ethanol). The curves are only guiding the eye.

Surprisingly, the worsened separation factor can also be a result of the PRO:XYL addition, since it has been evidenced that DES increased the free volume in CS films [13]. Tentatively, the usage of this hydrophilic PRO:GLU XYL revealed an impact in the selective properties of the CS membrane. Figure 7 represents the water and ethanol permeation as a function of temperature. It can be noticed that even if the separation factor was worsened, the DES still favored the transport of water while limiting the ethanol transport. Here, the solvent’s polarity becomes important for its own transport and separation [32]. Apart from that, the diffusion of water across the membranes can increase with an increase in feed water activity, which is ascribed to the plasticization effect on the membrane due to the increase in the free volume between the polymer chains, facilitating the diffusion of the molecules through the membrane matrix [41,42]. Fundamentally, the first transport resistance in a PV system is generally assumed to be in the membrane itself, according to its non-porous structure. The transport through a dense membrane can be described by the solution–diffusion model, in which the concentration dependence of solubility and/or diffusivity along with the coupling and plasticizing between components and the membrane are considered [43]. As for the diffusion coefficient, it is commonly affected by many factors, such as the coupling between diffusing components, component concentration, structure of the polymer, size of the permeating component, and degree of membrane swelling [31].

**Figure 8:**
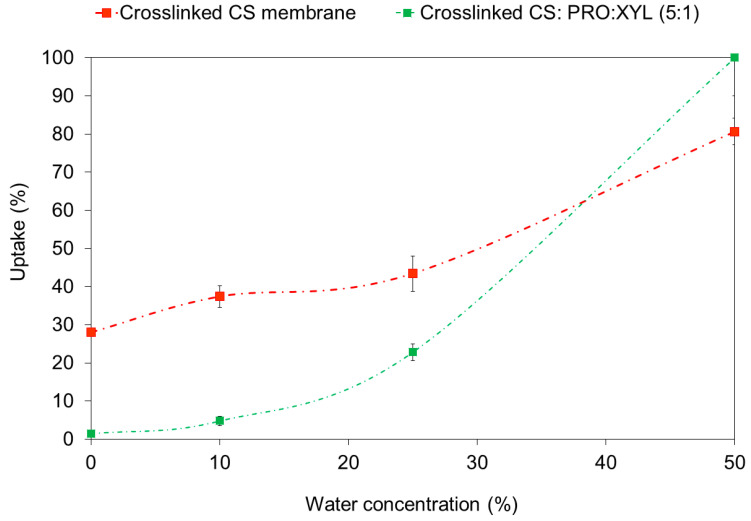
Uptake of CS-DES membranes at different water concentrations in ethanol (at room temperature). The curves are only guiding the eye.

As part of the membrane characterization, it was seen that both DES-free and DES-containing membranes display low uptake values at low concentrations of water in the ethanol mixtures, as displayed in Figure 8. Interestingly, the DES-containing membrane displayed a lower swelling degree than the pristine CS membrane. To some extent, when the water concentration increased, a higher swelling of the membranes was observed, ranging from 10 to 50 wt.% water concentration. Regarding the PRO: XYL addition, this resulted in a decrement in the solvent uptake in comparison with the DES-free membrane. According to a recent report [13], DES may confer stabilization to the CS membranes. It is important to address that the uptake decreases due to chemical crosslinking treatment, as there is a restriction in the motion of the polymer chain [44]. Since these membranes present a high degree of swelling, it is important to mention that the plasticization parameter of the membrane could be influenced by the temperature and concentration of the feed. Such a plasticization effect becomes relevant in the dehydration of alcohols using hydrophilic polymer membranes (such as PVA, CS, etc.) [42].

#### 3.2.2. Mass-Transfer Resistance Analysis

**Figure 9:**
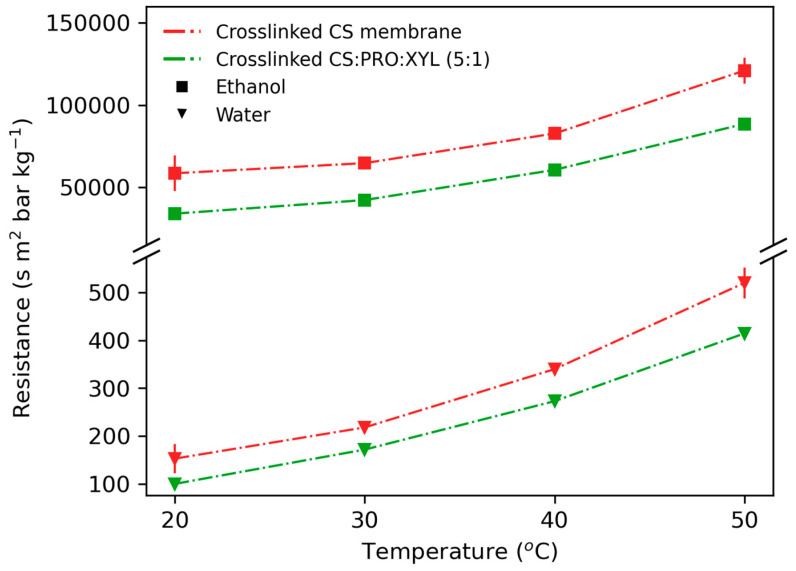
Comparison of ethanol and water mass-transfer resistance through membranes. Points connecting lines are to improve readability of the graph.

Figure 9 presents the comparison of the membrane resistance for the feed components. The contribution of the liquid phase to overall mass-transfer resistance was negligible. For the ethanol it was less than 0.01% and for the water it was less than 2%, regardless of the membrane material. The introduction of a DES reduced the membrane resistance toward water and ethanol molecules to a different extent. For ethanol, the mass-transfer resistance drop was almost 10% bigger than for water. Table 2 presents the pervaporation separation index (PSI), which defines the relative separation ability of a membrane as the product of the separation factor ( α ) and permeation rate [45]. The PSI was calculated by the following equation [46]:

(7) $$\mathrm{PSI}=J(\alpha_{W/E}-1)$$

The reduced resistance of the DES-containing membrane resulted in improved permeability of feed components and increased values of the pervaporation separation index (PSI). However, there is a trade-off between the membrane mass-transfer resistance and selectivity. As the permeability increases, the water/ethanol selectivity decreases. Nevertheless, it is still at a satisfactory level.

Selectivity depends also on temperature—as the temperature of the pervaporation system increases, the selectivity drops. For the CS:PRO:XYL membrane, it was reduced from 613 at 20 °C to 421 at 50 °C. The PSI value is rather stable regardless of the experimental temperature. The only exception was the value for the CS:PRO:XYL membrane at 20 °C, where the highest PSI value of 218 was observed.

Table 3 compares the CS:PRO:XYL membrane with other DES CS membranes reported in the literature. The data indicate that CS:PRO:XYL membranes have significantly lower apparent activation energy for permeate flux than other DES CS membranes (such as CS:PRO:GLU and CS:PCA:SULF). This is associated with the highest permeability of components and the highest total flux. Such a characteristic is beneficial from the point of view of pervaporation throughput; however, the CS:PRO:XYL membrane has the lowest selectivity among membranes presented in Table 3—it is two times lower. The difference in membrane selectivity can be linked to the difference in ethanol and water mass-transfer resistance (R_Ethanol/Water_), which is the lowest for the CS:PRO:XYL membrane. In contrast, the CS:PCA:SULF membrane has a similar R_Ethanol/Water_ value but almost two times higher selectivity and PSI values. Generally, the best pervaporation performance (the highest PSI value) was measured for the most hydrophilic CS membrane with the addition of CS:PRO:XYL DES, which results as the best CS-containing DES membrane for ethanol dehydration, also showing a more interesting flux than CS:PRO:GLU.

#### 3.2.3. Comparison of the Separation Performance of Crosslinked CS:PRO:XYL-Containing Membranes with the Literature

The separation performance of PV membranes is determined by the membrane properties, such as membrane material, nature, structure, etc., along with operational variables including feed composition, operating temperature, and pressure, among others [47]. In particular, the membrane structure is determined and tailored by the membrane preparation method [48]. As a starting point, we conducted a brief comparison of different membranes implemented for ethanol dehydration at similar separation conditions via PV, as enlisted in Table 2. For instance, the CS: PRO: XYL showed the best separation factor at 20 °C (ca. 613), while the highest total permeate flux (~0.467 kg m^−2^ h^−1^) was exhibited at 50 °C in both DES-free and DES-containing membranes while observing a selectivity decrement. The membrane containing the PRO:XYL demonstrated a significant improvement (around 25%) in flux compared with the bare CS membrane (see Table S1). Comparing with other DES CS membranes, CS:PRO:XYL membranes offered higher permeation than CS:PRO:GLU (~0.389 kg m^−2^ h^−1^) at the same operating conditions, as listed in Table 4 [24].

Crosslinked CS:PRO:XYL membranes presented better selectivity than crosslinked polyvinyl alcohol-filled graphene oxide, CS-doped H-ZSM-5, polyimide-filled ZIF-8, CS-filled TiO_2_, polyimide-loaded MSS-1, crosslinked CS:PRO:GLU and crosslinked polyvinyl alcohol-loaded amino functionalized ZIF-8. However, regardless of the inorganic nanomaterials loaded into the polymer membranes, the previous composites could offer even higher permeate fluxes than DES-containing membranes. Unfortunately, CS:PRO:XYL membranes did not exhibit more competitive separation properties than crosslinked sodium alginate-filled beta zeolite, NaP1 zeolite membranes and crosslinked CS-filled silica. Eventually, similar to crosslinked CS:PRO:GLU membranes, crosslinked CS:PRO:XYL membranes surpass the selective permeable trade-off of the bare CS membranes, with significant flux properties. Finally, the membranes developed in this study demonstrated a more attractive performance than commercial PV membranes, such as PVA composite membrane (from Deutsche Carbone AG/GFT) and PVA composite membrane (from PERVAP 2201, Sulzer Chemtech), used for ethanol purification.

## 4. Conclusions

In this work, we proposed a natural-based deep eutectic mixture, proline: xylitol, which is physically and chemically compatible with chitosan, for the fabrication of dense film membranes. Thanks to the full characterization, we confirm that the resultant crosslinked CS-hydrophilic L-proline: xylitol membranes are competent in terms of their miscibility into the biopolymer continuous matrix. In addition to this, such a new concept of composite membranes was tested for the purification of ethanol via PV technology, in which they benefited from the hydrophilic PRO:XYL mixture with a greater permeation rate. These flat sheet membranes demonstrated attractive separation that outperformed other recent composite membranes reported in the literature. The permeate flux offered by these membranes displayed a temperature dependence, in which the water permeation was fostered. This work shows a feasible approach to producing easy-to-prepare, less-costly, and eco-friendly membranes in the current framework of green chemistry. As a perspective, crosslinked CS-hydrophilic L-proline: xylitol membranes will be used as a main continuous phase for preparing mixed-matrix membranes filling nanosized inorganic materials.

## Figures and Tables

**Table 1:** Apparent activation energy values for total permeate, water and ethanol fluxes of the prepared membranes.

Membrane	Activation Energy Values (kJ/mol)		
	Total	Water	Ethanol
**Crosslinked CS**	5.15	5.07	10.27
**Crosslinked CS:PRO:XYL**	3.10	3.02	7.21

**Table 2:** Water/ethanol separation factor for studied membranes and corresponding pervaporation separation index.

T (°C)	Crosslinked CS		Crosslinked CS:PRO:XYL (5:1)	
	*α_W/E_*	*PSI* (kg m^−2^ h^−1^)	*α_W/E_*	*PSI* (kg m^−2^ h^−1^)
20	718.6 ± 15.3	166.8 ± 17	613.5 ± 19.0	218.2 ± 0
30	561.1 ± 23.8	168.5 ± 1	467.4 ± 0.7	178.3 ± 2
40	471.6 ± 4.4	159.5 ± 2	430.9 ± 4.4	181.2 ± 1
50	461.9 ± 2.2	171.7± 5	421.2 ± 1.9	196.2 ± 3

**Table 3:** Comparison of the DES-containing CS membrane parameters and separation performance at 30 °C.

Membrane	WCA (°)	E_app_ (kJ mol^−1^)	J (kg m^−2^ h^−1^)	R_Ethanol/Water_	S_W/E_	PSI (kg m^−2^ h^−1^)	Reference
CS:PRO:XYL	79	3.10	0.3823	246	246	218	This work
CS:PRO:GLU	50	5.63	0.3022	614	600	181	[24]
CS:PCA:SULF	100	5.54	0.3469	259	494	171	[27]

J—total permeate flux, R_Ethanol/Water_—ratio of the membrane resistance toward ethanol to the resistance toward water, S_W/E_—water/ethanol selectivity, PSI—pervaporation separation index, E_app_—apparent activation energy for total flux, WCA—water contact angle.

**Table 4:** Pervaporation separation of various polymer blends and composite membranes for ethanol dehydration and their comparison with crosslinked CS: PRO: XYL membrane performance.

Membrane Material	Filler Loading	Water Concentration	Operating Conditions	J (kg m^−2^ h^−1^)	Separation Factor (α)	Reference
Crosslinked CS:PRO:XYL	-	10 wt.%	20 °C, 1 mbar	0.356	613	This work
Crosslinked CS:PRO:XYL		10 wt.%	50 °C, 1 mbar	0.467	421	This work
Crosslinked PVA-filled GO	1 wt.%	10 wt.%	40 °C, 3 mbar	0.137	263	[49]
CS-filled H-ZSM-5	8 wt.%	10 wt.%	80 °C, 10 mbar	0.230	152	[50]
Crosslinked sodium alginate-filled beta zeolite	10 wt.%	10 wt.%	30 °C, 0.6 mbar	0.130	1600	[51]
Polyimide-filled ZIF-8	12 wt.%	10 wt.%	42 °C, 44 mbar	0.260	300	[52]
CS-filled TiO_2_	6 wt.%	10 wt.%	80 °C, 50 mbar	0.340	196	[53]
Polyimide-filled MSS-1	12 wt.%	10 wt.%	42 °C, 44 mbar	0.310	190	[52]
Crosslinked CS-filled silica	5 wt.%	10 wt.%	70 °C, 10 mbar	0.410	919	[54]
Crosslinked PVA-filled ZIF-8-NH_2_	7.5 wt.%	15 wt.%	40 °C, 1 mbar	0.120	200	[55]
NaP1 zeolite membranes	-	10 wt.%	75 °C, 4 mbar	0.45	200,000	[56]
PVA composite membrane (Deutsche Carbone AG/GFT)	-	10 wt.%	60 °C, 5 mbar	0.140	170	[57]
PVA composite membrane (PERVAP 2201, Sulzer Chemtech)	-	10 wt.%	60 °C, 10 mbar	0.100	100	[58]
Crosslinked CS:PRO:GLU	-	10 wt.%	20 °C, 1 mbar	0.242	1425	[24]
Crosslinked CS:PRO:GLU		10 wt.%	50 °C, 1 mbar	0.389	831.7	[24]

## Data Availability

Data are contained within the article.

## Supplementary Materials

The following supporting information can be downloaded at: https://www.mdpi.com/article/10.3390/membranes13040451/s1, Table S1: PV data of the pristine crosslinked CS and CS-DES membranes as a function of temperature (10:9 wt.% water-ethanol, pressure: 1 mbar).
