# Correction: Castro-Muñoz et al. Merging Proline:Xylitol Eutectic Solvent in Crosslinked Chitosan Pervaporation Membranes for Enhanced Water Permeation in Dehydrating Ethanol. *Membranes* 2023, *13*, 451

**DOI:** 10.3390/membranes16020066

**Published:** 2026-02-06

**Authors:** Roberto Castro-Muñoz, Maksymilian Plata-Gryl, Grzegorz Boczkaj

**Affiliations:** 1 Faculty of Civil and Environmental Engineering, Department of Sanitary Engineering, Gdansk University of Technology, 11/12 Narutowicza St., 80-233 Gdansk, Poland; 2 Tecnologico de Monterrey, Campus Toluca, Av. Eduardo Monroy Cárdenas 2000 San Antonio Buenavista, Toluca de Lerdo 50110, Mexico; 3 Advanced Materials Center, Gdansk University of Technology, 11/12 Narutowicza St., 80-233 Gdansk, Poland

In the original publication [1], there was a mistake in the legend for Figures 4 (AFM for Crosslinked CS membranes) and 5 (CA for Crosslinked CS). The mentioned parts of the figures were partially adapted from previously published studies; however, citations of the source articles were not included. The correct legends appear below.

**Figure 4.** AFM surface and 3D images (15 × 15 µm) of pristine CS (reproduced from [27]) and CS:PRO:XYL membranes.
**Figure 5.** CA values for pristine crosslinked CS (reproduced from [24]) and CS:PRO:XYL membranes.

The authors state that the scientific conclusions are unaffected. This correction was approved by the Academic Editor. The original publication has also been updated.
