# Supplementary material for: Merging Proline:Xylitol Eutectic Solvent in Crosslinked Chitosan Pervaporation Membranes for Enhanced Water Permeation in Dehydrating Ethanol
Source: Membranes (Basel). 2023 Apr 21;13(4):451. doi: 10.3390/membranes13040451 (PMC10146218; doi:10.3390/membranes13040451)
Supplement: Supplementary file 1 [file membranes-13-00451-s001.zip › membranes-2335566-supplementary.pdf]

Table S1. PV data of the pristine crosslinked CS and CS-DES membranes as a function of temperature (10:90 wt.% water-ethanol, pressure: 1 mbar).

Temperature	Total flux (kg m^{-2} h^{-1})	Separation factor (α)	Water partial flux (kg m^{-2} h^{-1})	Ethanol partial flux (kg m^{-2} h^{-1})
	**Crosslinked CS**			
**20°C**	0.2325±0.0528	718.6±15.3	0.2313±0.0521	0.0029±0.00062
**30°C**	0.3009±0.0100	561.1±23.8	0.2962±0.0098	0.0047±0.00027
**40°C**	0.3390±0.0143	471.6±4.4	0.3326±0.0140	0.0063±0.00032
**50°C**	0.3726±0.0275	461.9±2.2	0.3655±0.0270	0.0071±0.00056
	**Crosslinked CS:PRO:XYL**			
**20°C**	0.3564±0.0038	613.5±19.0	0.3513±0.0038	0.0005±0.00012
**30°C**	0.3823±0.0037	467.4±0.7	0.3750±0.0037	0.0072±0.00000
**40°C**	0.4217±0.0003	430.9±4.4	0.4131±0.0002	0.0086±0.00009
**50°C**	0.4671±0.0123	421.2±1.9	0.4574±0.0121	0.0097±0.00021
